# Psychometric properties of the AUDIT among men in Goa, India

**DOI:** 10.1016/j.ajp.2017.03.006

**Published:** 2017-10

**Authors:** Paige Endsley, Benedict Weobong, Abhijit Nadkarni

**Affiliations:** a Sangath, H No 451 (168), Bhatkar Waddo, Socorro, Bardez, Porvorim, Goa 403501, India; b 841/1, Alto Porvorim, Bardez, B/H Elctricty Dept, Bardez, Porvorim, Goa 403521, India; c London School of Hygiene and Tropical Medicine, London, UK; d Keppel Street, London WC1E 7HT, UK

**Keywords:** Alcohol use disorder, AUDIT, Validation, Goa, India

## Abstract

**Aims:**

The Alcohol Use Disorders Identification Test (AUDIT) is a 10-item screening questionnaire used to detect alcohol use disorders. The AUDIT has been validated in only two studies in India and although it has been previously used in Goa, India, it has yet to be validated in that setting. In this paper, we aim to report data on the validity of the AUDIT for the screening of AUDs among men in Goa, India.

**Methods:**

Concurrent and convergent validity of the AUDIT were assessed against the Mini International Neuropsychiatric Interview (MINI) and World Health Organisation Disability Assessment Scale (WHODAS) for alcohol abuse, alcohol dependence, and functional status respectively through the secondary analysis of data from a community cohort of men from Goa, India.

**Results:**

The AUDIT showed high internal reliability and acceptable criterion validity with adequate psychometric properties for the detection of alcohol abuse and dependence. However, all of the optimal cut-off points from ROC analyses were lower than the WHO recommended for identification of risk of all AUDs, with a score of 6–12 detecting alcohol abuse and 13 and higher alcohol dependence.

**Conclusions:**

In order to optimize the utility of the AUDIT, a lowered cut-off point for alcohol abuse and dependence is recommended for Goa, India. Further validation studies for the AUDIT should be conducted for continued validation of the tool in other parts of India.

## Highlights

• The Konkani AUDIT showed high internal reliability and acceptable criterion validity.
• All optimal cut-off scores were found to be lower than WHO-recommended scores.
• Lowered cut-off points for alcohol abuse/dependence are recommended in Goa, India.
• Lowered cut-off points could have implications for treatment/seeking interventions.

## 1 Introduction

Alcohol Use Disorders (AUD) encompass a range of conditions related to excessive alcohol consumption and is recognized by the World Health Organisation (WHO) as a unique disorder: with hazardous, harmful, and dependent drinking comprising the progressively more serious forms of the disorder (Reid et al., 1999). AUDs account for a significant global burden of disease, injury, economic and social cost (Rehm et al., 2009, WHO and Team, 2014). The large societal cost of AUDs is not limited to healthcare costs, but also include unmeasured costs related to social harm, loss of productivity and direct law enforcement costs. Due to the large societal cost and burden of AUDs globally, appropriate screening tools are required to properly identified AUDs. Screening tools are particularly useful in low resource settings where efficiency is required in time and human resources when it comes to the detection of health problems.

The Alcohol Use Disorders Identification Test (AUDIT), developed by the WHO for the early detection of hazardous and harmful alcohol consumption, is one of the most widely used screening tools for the detection of AUD (Saunders et al., 1993). It is also able to detect patients with alcohol dependence, making it a more versatile and useful screening tool compared to the 4-item CAGE questionnaire (Ewing, 1984), and the 25-item Michigan Alcoholism Screening Test (Selzer, 1971). Whilst acknowledging the cross-national standardization of the AUDIT as a notable strength in the field of cross-cultural psychiatry, we identify with the argument by Altman and Bland that a tool is only valid in the setting in which it is valid (Altman and Bland, 1994).

The AUDIT has been previously validated in only two settings in India; a community-based sample in North India (Pal et al., 2004) and a clinic sample in Bangalore (Carey et al., 2003). However, we identify important concerns with the previous validation studies (Table 1). In the community study the criterion measure was not a diagnostic tool, but another screening tool, Short Michigan Alcoholism Screening Test (SMAST) (Pal et al., 2004). Further to this, in an attempt to increase the psychometric properties of the AUDIT, the authors have only selected participants with hazardous drinking (identified as AUDIT score 8 and above), thereby limiting the generalizability of the validated AUDIT, and more importantly defeating the purpose of cross-cultural adaptation of tools, where it is likely that previously ascertained cut-offs may perform differently in different cultural settings. In the clinic based study, apart from the fact that there was no gold standard criterion, we argue that validity studies from high prevalence settings may not generalize to the community as the process of seeking healthcare, the interaction with clinicians, and relatively high proportions of more severe disorders may all lead to bias (Carey et al., 2003). To our knowledge the AUDIT has not been previously validated against an established gold standard measure in a community-based population anywhere in India.

**Table 1:** Validation studies of the AUDIT in India.

Study	Key psychometrics	Sample	Gold standard criterion	Suggested cut-off scores
Pal et al. (2004)	Internal consistency Interscale correlations Sensitivity (Abuse: 85.3, Dependence: 69.4) Specificity (Abuse: 89.4, Dependence: 87.5) ROC Analysis (AUC = 0.883)	Community outreach sample (*n* = 200) and de-addiction center sample (*n* = 97)	Short Michigan Alcoholism Screening Test (13 item questionnaire differentiating between borderline harmful drinking and potential alcohol abuse)	Alcohol Abuse: 16 Alcohol Dependence: 24
Carey et al. (2003)	Feasibility Factor structure Reliability (alpha = 0.94) Validity Utility	Admissions to Psychiatric Hospital in Bangalore (*n* = 1349)	Clinician diagnosis at discharge, no gold standard criterion	Not applicable

The aim of this study was to determine the criterion and concurrent validity, scale reliability and psychometric properties of the local language (Konkani) version of the AUDIT for the screening of AUDs among men in Goa, India. Despite the sample consisting of only men, the unique context surrounding alcohol use within India justifies this homogeneity, as abstinence rates are high in women, due to the confluence of strong cultural and taboo factors (Benegal, 2005, Rehm et al., 2009).

## 2 Methods

### 2.1 Setting

This sub-study is a part of a large community-based cross-sectional study conducted in Goa, which has a population of just over 1.4 million, 62% of whom live in urban areas (Chandramouli and India. Office of the Registrar General & Census Commissioner., 2011).

### 2.2 Participants and follow up procedures

Participants were adults aged 18–49 years and residing in the following study sites between 2006 and 2008 (baseline survey), and who completed a follow-up survey 6–8 years later: urban (two beach areas popular among tourists and one typical commercial and residential area) and rural areas (six contiguous villages) of Northern Goa (Pillai et al., 2013). A two-stage probability sampling procedure, based on electoral rolls, was employed to determine the population-based sample. The participants were selected at random from those with eligible ages within the randomly selected households. Refusal rates for randomly selected households were 1.5%.

At a follow-up from September 2012 to September 2014, a range of self-reported outcomes were measured on the baseline cohort, including AUDIT, MINI, and WHODAS. All consenting participants were administered the self-report questionnaire by trained research workers. The research workers were blind to any AUD status gathered from baseline, and the data analyzed here was taken only from the follow-up measurements. Quality control was conducted by re-interviewing 10% randomly selected participants by the research coordinator and random visits by the research coordinator to directly observe the research workers.

### 2.3 Ethics

Ethical approval was obtained from the Sangath Institutional Review Board (IRB), Ethics Committee of the London School of Hygiene and Tropical Medicine (LSHTM) and the Indian Council of Medical Research. Each research worker completed the NIH Protecting Human Research Participant online course. Participants diagnosed with AUD or Common Mental Disorder (CMD defined as depressive and anxiety disorders) were offered further free clinical assessment and treatment by a psychiatrist.

### 2.4 Assessments

#### 2.4.1 Gold standard criterion measure

##### 2.4.1.1 MINI

The Mini International Neuropsychiatric Interview (MINI) was used to identify current alcohol abuse and alcohol dependence (Lecrubier et al., 1997). The MINI is a short diagnostic structured interview to explore 17 disorders according to Diagnostic and Statistical Manual IV-TR diagnostic criteria. It allows for administration by non-specialized interviewers. Interviews were conducted using paper and pencil with diagnosis assessed following a structured algorithm. Automatic exclusion of a diagnosis of alcohol abuse or dependence was made if the respondent answered no to the question “In the past 12 months, have you had 3 or more alcoholic drinks within a 3 hour period on 3 or more occasions?” Alcohol abuse was diagnosed if a positive response was given to any one of four questions regarding alcohol consumption; alcohol dependence was diagnosed if a positive response was given to any three of seven questions regarding alcohol consumption.

#### 2.4.2 Concurrent validity measure

##### 2.4.2.1 WHODAS

The WHO Disability Assessment Schedule (WHODAS) is a 12-item questionnaire for measuring functional impairment over the previous 30 days. In addition, two items assess number of days the person was unable to work in the previous 30 days. The WHODAS has uniform response options ranging from 0 to 4, and provides a continuously distributed summed up score of up to 48. In the present analyses, the WHODAS was used to assess health and general disability and functional status of participants. The WHODAS assesses disability in a range of functions including: standing, walking, concentrating, learning, household responsibilities, maintaining personal hygiene, dressing, social relationships, work, and emotions due to health problems.

#### 2.4.3 Test measure

##### 2.4.3.1 AUDIT

The AUDIT is a 10-item screening questionnaire originally developed by the WHO for the early detection of hazardous and harmful alcohol consumption (alcohol abuse), including alcohol dependence in primary health care (Ustun et al., 2010). Each item is scored on a scale of 0 to 4, and generates a continuously distributed total score ranging from 0 to 40. Based on its initial validation, summed up scores of 8–15, 16–19, and 20 or more, represent probable diagnosis of hazardous use, harmful use, and alcohol dependence respectively (Babor et al., 2001). In its initial development, it recorded sensitivities in the mid 0.90 and specificities averaging 0.80 for various degrees of problematic drinking at a cut-off of 8 (Saunders et al., 1993). AUDIT items 2 and 3 assess alcohol consumption based on ‘standard drinks’. This involved converting volumes of local drinks to the equivalent of a ‘standard drink’. For example, 0.5 pegs (30 ml) of ‘*caju feni’* (a local gin) is equivalent to 1 ‘standard drink’ (Nayak et al., 2008). All responses from interviews were collected on paper questionnaires in which the relevant tools were translated into the vernacular using a protocolised translation and back-translation procedures followed by piloting for language.

### 2.5 Statistical methods

The psychometric properties of the AUDIT were determined using Receiver Operating Characteristics (ROC) analysis with the MINI case criterion as the gold standard in order to generate the area under the curve and the optimal cut-point. The ROC analysis also yielded sensitivity and specificity estimates, including likelihood ratios (+/−) at that cut-point. In addition to this, we estimated Youden's index associated J point, a measure of overall test performance maximizing (sensitivity + specificity − 1), in order to compare our validity coefficients directly with those reported in other similar studies (Fluss et al., 2005). Agreement between the test cut-point and the gold standard was assessed using Cohen's Kappa. The internal scale consistency of the measures was ascertained by Cronbach's alpha. Concurrent validity of the AUDIT was assessed with Pearson's correlation coefficient for the correlation with the WHODAS functional disability and number of disability days. All analyses were conducted using STATA 13.

## 3 Results

The analysis involved 600 men with data on both the AUDIT and the MINI. Mean age at baseline was 32.7 years (range 18–49, SD 0.34). According to the MINI rating of current alcohol dependence and alcohol abuse, 62 men (10.3%) and 164 (27.3%) respectively met the gold standard criterion. The mean AUDIT test score found was 6.78 (SD 6.36), with a median score of 5 (IQR 2–9).

**Fig. 1:**
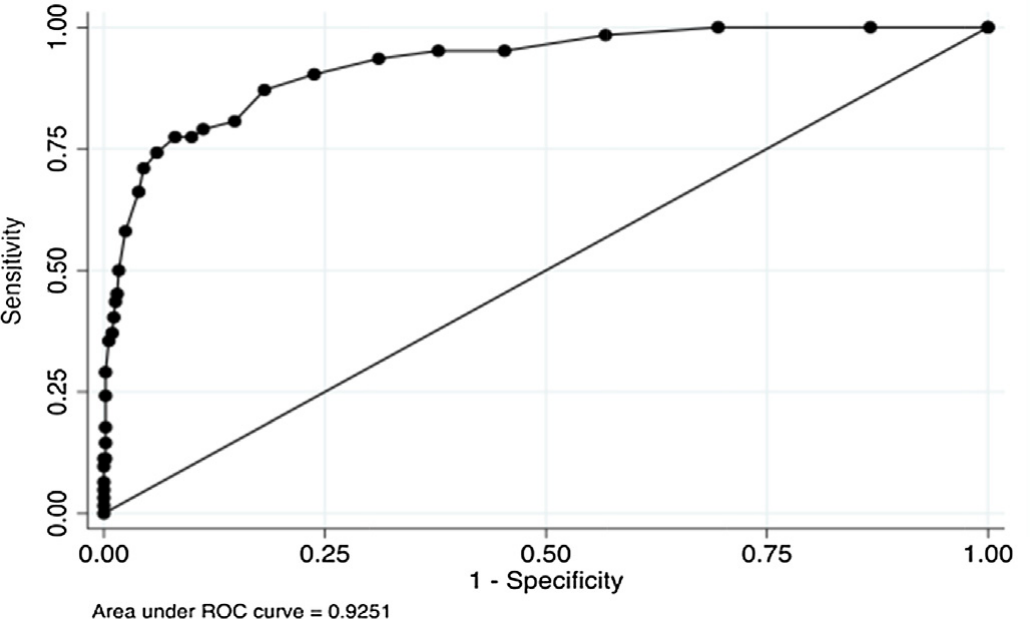
Receiver operating curve for AUDIT using MINI criteria for alcohol dependence. Area under ROC curve = 0.9251.

**Fig. 2:**
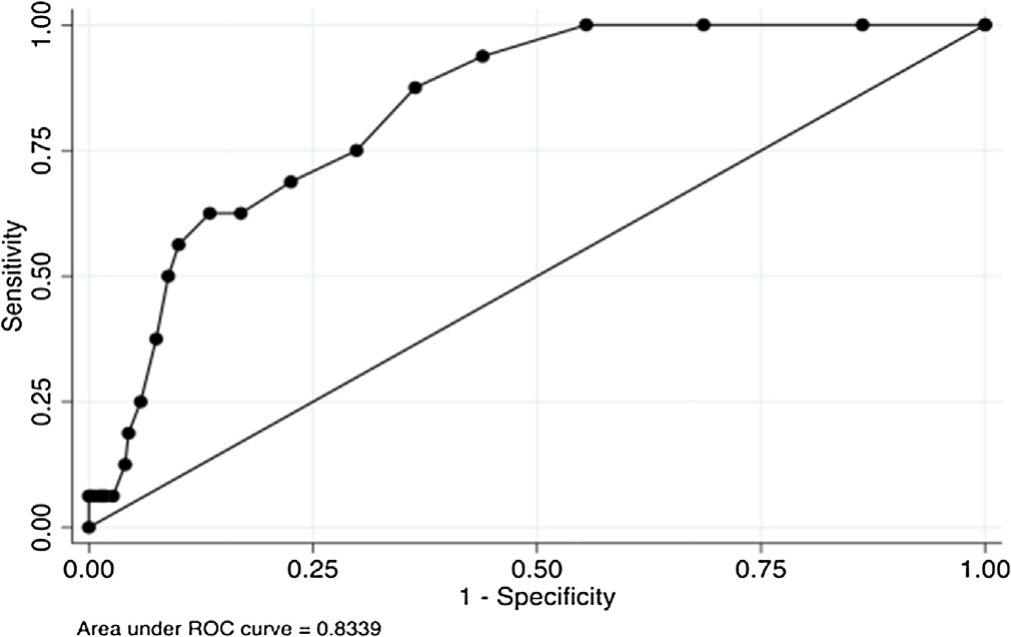
Receiver operating curve of the AUDIT using MINI criteria for alcohol abuse. Area under ROC curve = 0.8339.

The main findings on the test score distribution, reliability and validity are summarized in Table 2. The internal consistency of the AUDIT was strong (0.84), and the agreement with the gold standard criterion was modest for alcohol dependence (0.57) but weak for alcohol abuse (0.12). In terms of criterion validity, the area under the ROC curve was 0.93 for alcohol dependence and 0.83 for alcohol abuse (Fig. 1, Fig. 2). The corresponding optimal cut-points selected for the best sensitivity and specificity were 13 and 6 for alcohol dependence and abuse respectively. At these cut-points, 93 out of 600 men (15.5%) met criteria for probable alcohol dependence, and 171 out of 600 men (28.5%) met criteria for probable alcohol abuse. The Pearson's correlation coefficient for probable alcohol dependence and WHODAS functional disability was stronger (0.65) than with alcohol abuse (0.27).

**Table 2:** Test scale distribution, reliability, and validity.

Test scale	AUDIT	
*Scale distribution*		
Mean	6.78 (sd = 6.36)	
Median (IQR)	5 (2–9)	
*Scale Reliability*		
Cronbach's alpha	0.84	
Kappa	0.57 (Dependence); 0.12 (Abuse)	
*Criterion validity against MINI gold standard*	Dependence	Abuse
AUROC	0.93	0.83
Optimal cut-point	13	6
*At this cut-point*		
Sensitivity	0.77	0.87
Specificity	0.91	0.63
LR+	9.61	2.40
LR−	0.25	0.19
Youden's Index	0.69	0.51
Prevalence	15.5% (93/600)	28.5% (171/600)
*Concurrent validity*		
MINI total score		
Dependence	0.65	
Abuse	0.27	
WHODAS disability	0.22	
Dependence	0.24	
Abuse	0.05	
Disability days	0.21	
Dependence	0.25	
Abuse	0.01	

In Table 3, the criterion validity was assessed using the WHO standards for optimal cut-points, 20 and 16 for alcohol dependence and abuse respectively. At these cut-points, 34 out of 600 (5.67%) met criteria for probable alcohol dependence, and 31 out of 600 (5.17%) met criteria for probable alcohol abuse.

**Table 3:** Criterion validity against MINI gold standard using WHO cut-points.

	Dependence	Abuse
WHO cut-point	20	16
*At this cut-point*		
Sensitivity	0.44	0.26
Specificity	0.98	0.97
LR+	33.22	9.19
LR−	0.57	0.76
Prevalence	5.67% (34/600)	5.17% (31/600)

## 4 Discussion

We set out to assess the reliability and validity of the AUDIT for the detection of alcohol dependence and alcohol abuse among men in Goa, India. The results point to the AUDIT being both reliable and valid in detecting dependence and abuse among men in Goa, India with reasonably robust psychometric properties in detecting both outcomes. In this study, the AUDIT proved to have high internal reliability, with a Cronbach's coefficient of 0.84, which is also consistent with previous findings. In two reliability generalization analyses using studies prior to and post the year 2000, the median reliability coefficients were found to be 0.81 and 0.83 (Shields and Caruso, 2003, Reinert and Allen, 2007). The optimal cut-off score that maximizes both sensitivity and specificity of the AUDIT in identifying alcohol dependence among men in this setting is 13. At this cut-point, the AUDIT recorded a relatively modest sensitivity (77%) and high specificity (91%). The ideal cut-off score for identifying alcohol abuse among men was found to be 6, with high sensitivity (87%) and slightly poor specificity (63%).

These cut-offs and diagnostic properties are very similar to those found in other studies. A validation of the Nepali version of the AUDIT suggested cut-offs of 5 and 11 for hazardous use and alcohol dependence respectively (Pradhan et al., 2012). However, sensitivity and specificity estimates here are slightly poorer than those reported in Nepal. Similarly, a study conducted in Switzerland found optimal cut-offs ranging from 10 to 13 for identifying alcohol dependence with slightly stronger diagnostic properties than those here (Daeppen et al., 2000). Further, an epidemiological survey conducted in Tibet using the Tibetan version of the AUDIT found appropriate cut-offs of 10 and 13 for alcohol abuse and dependence respectively (Guo et al., 2008). For both of these cut-offs, sensitivity and specificity were all over 84%, comparable to the results found here. A validation of the AUDIT among Nigerian students also found similar cut-offs of 7 and 9 for alcohol abuse and dependence, with all diagnostic properties greater than 86% (Adewuya, 2005). Finally, a validation study of the French version of the AUDIT found that among men, the ideal cut-off for alcohol dependence was also 13 with similar psychometric properties to those presented here (Gache et al., 2005). All of these findings suggest cut-off scores much lower than those recommended by the WHO.

Concurrent validity for the AUDIT against the MINI and WHODAS for disability days experiencing disability was relatively low, except for alcohol dependence as judged by the AUDIT and MINI. However, a trend was evident, as there was a much higher correlation between identification of dependence by the AUDIT and MINI and WHODAS than any correlation between those tests and identification of alcohol abuse by the AUDIT. Although the correlation was higher for dependence than abuse, the low concurrent validity values allow us to conclude that AUDIT scores do not correlate with disability associated scores from the WHODAS, and perhaps that the AUDIT test does not adequately assess disability associated with alcohol consumption. Furthermore, these results suggest that the diagnosis of alcohol abuse on the MINI does not correlate with a positive alcohol abuse screening from the AUDIT.

### 4.1 Clinical implications

While it has been suggested that cultural differences may influence the threshold for alcohol use disorders, the AUDIT still proves to be reliable and valid for AUD identification in Goa, India. The differences seen here in optimal cut-off scores for this setting versus those recommended by the WHO are not surprising, and it is likely that there is a cross-cultural difference in the threshold for alcohol use disorders identification. We argue therefore that maintaining the WHO recommended cut-offs in our setting may result in a differential misclassification bias, with an increase of false negatives as evidenced through the higher likelihood ratio negative when using WHO cut-points. This could have huge implications for health-seeking and treatment interventions; patients with a score between 13 and 19 in our setting meet criteria for probable alcohol dependence, and should referred to a specialist for diagnostic evaluation and treatment. Further to this, the relatively high specificity of the AUDIT in ruling out alcohol dependence coupled with the adequate sensitivity in ruling in alcohol abuse make the AUDIT a very useful tool in our setting. It is likely to be effective if used as a screening tool or program in primary care as given the majority of patients are likely to present with alcohol abuse, they will be identified as such and no further diagnostic tests will be required, thus saving the additional costs for diagnostic tests (which may not be readily available in low- and middle-income country settings as a result of human resource challenges). Conversely, the ability of the AUDIT to rule out alcohol dependence (who are likely to be few relative to alcohol abuse patients) means the few false positives can lend themselves to a diagnostic test, thus reducing the burden on the health system.

### 4.2 Limitations

The main limitation with this study is the inability to establish criterion validity with a clinician's diagnosis, which would have been the optimal gold standard. Having said this we are aware that lay interviewers can be trained to administer fully structured clinical interviews such as the MINI (Prince, 2003). We also recognize the potential for interviewer bias in this sub-study as the same research worker administered both the AUDIT and the MINI. However, the effect of this bias in the interpretation of the findings of this sub-study is lessened by the fact that the research worker was blind to the research question of this sub-study. Further, we were unable to assign random order to the completion of each test, which may have introduced order effects into our results. Finally, the inability of the MINI to identify hazardous drinking did not allow us to determine the diagnostic properties of the AUDIT for the detection of hazardous drinking.

## 5 Conclusions

To the best of our knowledge, this is the first large community-based study on the psychometric properties of the AUDIT among men in India. The results from this study show that among a sample of men from Goa, India the WHO recommended cut-off points for identification of AUDs may be too high and may not be generalizable in this setting. Lowering the cut-offs for identification of AUDs using the AUDIT in this setting may allow for a more responsive screening tool. The results here prove that the Konkani version of the AUDIT shows considerable promise, but its utility and validity in other parts of India requires further investigation.

## Funding

This work was supported by the Wellcome Trust Research Training Fellowship to Abhijit Nadkarni [grant number WT093897MA].

## Conflict of interest statement

None declared.
